# Electroacupuncture Alleviates 46-Trinitrobenzene Sulfonic Acid-Induced Visceral Pain via the Glutamatergic Pathway in the Prefrontal Cortex

**DOI:** 10.1155/2023/4463063

**Published:** 2023-01-19

**Authors:** Hao Jiang, Rongrong Li, Fan Zhang, Feini Zhou, Jiangnan Lin, Ning Kong, Haitao Chen, Lingnan Guo, Chenxiao Ye, Fuhao Li, Maosheng Xu

**Affiliations:** ^1^The First School of Clinical Medicine of Zhejiang Chinese Medical University, Hangzhou, Zhejiang 310053, China; ^2^Department of Radiology, The First Affiliated Hospital of Zhejiang Chinese Medical University, Hangzhou, Zhejiang 310003, China; ^3^Key Laboratory of Digestive Pathophysiology of Zhejiang Province, the First Affiliated Hospital of Zhejiang Chinese Medical University, Hangzhou, Zhejiang310006, China; ^4^The Third School of Clinical Medicine (School of Rehabilitation Medicine) of Zhejiang Chinese Medical University, Hangzhou, Zhejiang 310053, China; ^5^The Cancer Hospital of the University of Chinese Academy of Sciences (Zhejiang Cancer Hospital), Hangzhou, Zhejiang 310022, China; ^6^Institute of Basic Medicine and Cancer (IBMC), Chinese Academy of Sciences, Hangzhou, Zhejiang 310022, China

## Abstract

Visceral pain caused by inflammatory bowel disease (IBD) greatly diminishes the quality of life in affected patients. Yet, the mechanism of how IBD causes visceral pain is currently not fully understood. Previous studies have suggested that the central nervous system (CNS) and gut-brain axis (GBA) play an important role in IBD-inducing visceral pain. As one of the treatments for IBD, electroacupuncture (EA) has been used to treat various types of pain and gastrointestinal diseases in clinical practice. However, whether EA relieves the visceral pain of IBD through the gut-brain axis has not been confirmed. To verify the relationship between visceral pain and CNS, the following experiments were conducted. ^1^H-NMR analysis was performed on the prefrontal cortex (PFC) tissue obtained from IBD rat models to determine the link between the metabolites and their role in EA treatment against visceral pain. Western blot assay was employed for detecting the contents of glutamate transporter excitatory amino acid transporters 2 (EAAT2) and the glutamate receptor N-methyl-D-aspartate (NMDA) to verify whether EA treatment can alleviate neurotoxic symptoms induced by abnormal increases of glutamate. Study results showed that the glutamate content was significantly increased in the PFC of TNBS-induced IBD rats. This change was reversed after EA treatment. This process was associated with increased EAAT2 expression and decreased expression of NMDA receptors in the PFC. In addition, an increase in intestinal glutamic-metabolizing bacteria was observed. In conclusion, this study suggests that EA treatment can relieve visceral pain by reducing glutamine toxicity in the PFC, and serves an alternative clinical utility.

## 1. Introduction

Inflammatory bowel diseases (IBD), including Crohn's disease (CD), and ulcerative colitis (UC), describe a subset of chronic inflammatory diseases [1]. Previous epidemiological studies have shown that the incidence of IBD increases year by year [2]. The main symptoms of IBD include visceral pain, diarrhea, absorptive disorders, weight loss, and fecal incontinence [3, 4]. Visceral pain is one of the most common and bothersome symptoms of IBD [5]. Even with well-controlled IBD, visceral pain may persist for up to a year after relevant treatment is initiated [6–8]. Visceral pain harms the health-related quality of life [8] and imposes a huge economic burden on patients and the healthcare system [9]. Therefore, it is particularly important to find an effective method to relieve the symptoms of visceral pain in IBD patients.

The current mainstream view of IBD-related visceral pain is that it is derived from dysregulation in the central nervous system (CNS) and gut-brain axis (GBA) [10]. The visceral pain state can be built by neurovisceral interactions [11]. Inflammation in IBD sensitizes sensory nerves and creates the sensation of pain. As such, the synergistic effect between the brain and the intestine provides an important basis for visceral pain. Numerous medications have been used to treat visceral pain, such as opioids, anesthetics, and nonsteroidal anti-inflammatory drugs. However, the inappropriate use of drugs often results in a reduction in efficacy, drug resistance, and adverse effects [12–16].

As an alternative to traditional Chinese medicine (TCM), nowadays, the use of acupuncture has been advocated in the treatment of various inflammatory disorders [17]. Acupuncture works by using small needles to stimulate specific acupuncture points in the body [18]. It has been widely used to treat gastrointestinal diseases such as CD [19], UC [20], and irritable bowel syndrome (IBS) [21]. Previous studies have shown that electroacupuncture can improve intestinal function by downregulating the expression of 5-HT1A receptor (5-HT1AR) and C-FOS proteins [22], and can inhibit the expression of purinergic receptor P2X3 (P2X3) to achieve an analgesic effect on inflammatory pain [23].

Zusanli (ST36) is one of the key acupuncture points in the treatment of gastrointestinal diseases [24]. Specifically, ST36 is located on the anterior tibial muscle group between ST35 and ST41 [25]; it functions to reduce inflammation [26], enhance immunity [27], and promote the function of the gastrointestinal tract [28]. Shangjuxu (ST37), another key acupuncture point, helps in improving intestinal permeability [29] and enhancing immune function through the toll-like receptor 4 (TLR4) pathway [30]. Previous studies have shown that acupuncture of the ST36 and ST37 sites can reduce cell apoptosis and restore the intestinal barrier function and thereby protecting the intestinal mucosa [31, 32]. Therefore, in this study, ST36 and ST37 were selected as the therapeutic acupoints for IBD rats.

Glutamate is the most abundant excitatory neurotransmitter in CNS [33]. Glutamic neurons release glutamic acid, which induces excitatory neurotoxicity and promotes pathologic pain [34]. N-methyl-D-aspartate (NMDA) is an important receptor in glutamate signaling [35]. The increase of extracellular glutamate can lead to the activation of NMDA, resulting in the long-lasting excitability of neurons [36].

While EA can alleviate nociception via the inhibition of the glutamate transporter [37] and relieve visceral pain caused by IBD [38], the effect of EA on the glutamic neurons in the prefrontal cortex (PFC) has not been reported. Different from previous studies, in this study, we pioneered a link between the glutamatergic system of the prefrontal lobe and the EA treatment of IBD-induced visceral pain and tried to prove that such visceral pain can be relieved through EA treatment via the reduction of the glutamate toxicity in PFC. We used 4,6-trinitrobenzenesulfonic acid (TNBS) to create models of IBD in rats. Metabolic studies were used to measure the glutamate content in the PFC. Western blot assay was used to investigate the effect of EA on TNBS rats by analyzing the glutamic neurons in the PFC. Finally, 16S rDNA gene sequencing and enzyme-linked immunosorbent assay (ELISA) were used to determine the changes in the digestive tracts.

## 2. Materials and Methods

### 2.1. Experimental Animals

Male Sprague-Dawley (SD) rats were purchased from the Shanghai Sippr-BK Laboratory Animal Co., Ltd. and studies adhered to the guidelines for experimental pain in animals published by the International Association for the Study of Pain (LASP). Rats were housed at the Zhejiang Chinese Medical University of Animal Care Center at 24 ± 2°C, a humidity of 55 ± 10%, with low noise and a 12 h/12 h alternating light-dark cycle. The average weight of rats was about 220-240 g. The rats were fed for one week after arrival in the laboratory with both food and water. Before the start of the study, animals fasted for one day. This experiment was supervised and approved by the Experimental Animal Ethical Committee of the Zhejiang Chinese Medical University (Approved No. of ethics committee: ZSLL-2018-014). A total of 24 SD rats were divided randomly into three groups; the control, TNBS, and TNBS + EA groups (TNBS rats treated with EA treatment) with 8 rats in each group.

The body weights of rats before TNBS injection were measured as the baseline. Then, the weight of each group was tested once daily for 14 days. Colorectal distension (CRD) test was performed on the 14^th^ day following the administration of TNBS. On the 15^th^ day after the initiation of TNBS, the rats were sacrificed for organ harvesting. Based on the weight data recorded on the last day, rats were anesthetized with 3% pentobarbital. The rats were euthanized after taking blood from the celiac vein. PFC, colon tissue, feces in the cecum, and serum were collected and stored at -80°C. The colon length of each rat was recorded.

### 2.2. Modeling Method of the IBD Rat Model

We modeled the experimental rats one day before the start of the animal experiment. The IBD model induced by TNBS was implemented in male rats as previously described [39]. The rat model of inflammatory bowel disease was carried out according to the article by He et al. with appropriate adjustments [40]. A TNBS-ethanol mixture was prepared by mixing one volume of 5% TNBS solution (Sigma-Aldrich, St. Louis, MO, USA to be determined, origin 50 g/L) and one volume of 75% ethanol (anhydrous ethanol and PBS solution 3: 1 configuration) was used.

The rats were anesthetized with 3% pentobarbital and a catheter was inserted (8 cm to the anal verge) through the anus. The TNBS solution was infused at a dose of 3.2 mL/kg. Afterward, the plastic sheath was removed from the gut and the rat was positioned cephalocaudally for one minute to ensure that the TNBS-ethanol mixture remains completely in the gut.

### 2.3. EA Treatment

Rats in the TNBS + EA group were treated with EA for 20 minutes every day for 14 days after modeling. The rats were restrained before EA treatment but not given any anesthetics. To reduce the interference of external factors, the rats in the control group and TNBS group also wore shackles and were given a sham acupuncture treatment. Bilateral ST36 and ST37 were chosen for EA treatment. The insertion depth was set at 10 mm. Then the needles were stimulated by an EA treatment (Hwato SDZ-IIB, Suzhou Medical Supplies Factory Co., Ltd., Suzhou, China) with a frequency of 5 Hz and an intensity of 2 mA.

### 2.4. Colorectal Distention (CRD) Test

On the 14th day of the animal study, we performed colon dilatation experiments. Visceral hypersensitivity (VH) was hypothesized to be the underlying mechanism of visceral pain [5, 41]. We measured the mechanical threshold, which was inversely associated with VH, to reflect visceral pain using CRD. The abdominal retraction was observed in the CRD test to assess mechanical thresholds.

After 30 min of acclimation to the experimental environment, a straitjacket was placed on the experimental subject. The lower abdomen of the rats was exposed. A catheter with a balloon was inserted into the colon until the balloon was located intra-anally 1 cm proximal to the anus. The balloon was slowly inflated until the lower abdomen rested against the floor. The abdominal retraction and the air volume were recorded. The test was performed three times at an interval of 5 min. The control, TNBS, and TNBS + EA groups all participated in this experiment.

### 2.5. Histopathological Assessment

Fresh colon samples of 1 cm in length were placed in formalin for fixation. The colon samples were sectioned with a microtome at 4 *μ*m-thick after embedding in paraffin. Then, the section was stained with hematoxylin and eosin (HE). Light microscopy was used for observing the intestinal tissue structure, intestinal mucosa, and inflammatory cell infiltration.

### 2.6. Western Blot Analysis

The tissue obtained from the PFC was mechanically lysed before being placed in RIPA buffer. The supernatant was mixed with 5 times the volume of loading buffer at a 4: 1 ratio and boiled for 10 minutes. The protein was then separated in a 10% SDS-polyacrylamide gel electrophoresis (SDS-PAGE). Afterward, the protein mixture was filtered using a 0.45 *μ*m PVDF membrane (Millipore, Massachusetts, USA) and blocked with protein-free rapid blocking buffer (EpiZyme, China). Membranes were stored at 4°C overnight along with the primary antibody. Afterward, samples were incubated with horseradish peroxidase-coupled secondary antibodies for 1 hour at room temperature and developed using ECL chemiluminescence substrate. Primary antibodies used were anti-NMDA (5704, Cell Signaling; 1: 1,000), anti-EAAT2 (20848, Cell Signaling; 1: 1,000), and anti-GAPDH (2118, Cell Signaling; 1: 1,000), anti-*β*-actin (, Cell Signaling; 1: 1,000).

### 2.7. 16S rRNA Miseq Sequencing and Bioinformatic Analysis

The E.Z.N.A. ®Stool DNA Kit (D4015, Omega, Inc., USA) was used to extract the total microbial genomic DNA from 200 mg of feces in each rat according to the manufacturer's instructions. The V3-V4 region of the bacterial 16S rRNA gene was used as the forward primer 341F (5′-CCTACGGGNGGCWGCAG-3′) and the reverse primer 805R (5′-GACTACHVGGGTATCTAATCC-3′). The sequence was obtained by the Illumina MiSeq platform. AMPure XT beads (Beckman Coulter Genomics, Danvers, MA, USA) were used to purify the mixture of PCR products [42]. The purified product was quantified by Qubit (Invitrogen, USA). Illumina NovaSeq platform was used to analyze the 16S rRNA gene sequences. Sequencing libraries were generated by the KaPA Library Quantification Kit. The sequences with a similarity greater than 97% were clustered into the same operational taxonomic units (OTUs). QIIME2 software was used for analysis.

### 2.8. The Sample Preparation for ^1^H-NMR Analysis

To reconstitute the aqueous phase, the PFC tissue (100 mg for each rat) was moved to 0.8 mL of 10% D2O phosphate buffer pH 7.4 (containing 0.05% 3-trimethylsilyl-propionate-d4; SIGMA, USA). The tissues were homogenized at 60 Hz for 2 cycles (1 cycle including 45 s crash and 15 s rest). Samples were centrifuged at 13,000 RPM at 4°C for 10 min. The supernatant was collected and transferred into 5 mm NMR tubes for NMR spectroscopic analysis.

PFC samples were selected to be analyzed by a Bruker 600 MHz AVANCE III spectrometer equipped with a 5 mm-BBFO probe. The temperature was set at 25°C and a lock was performed on the D2O signal. Each sample was shimmed and calibrated to the proton pulse before data acquisition. The NOESYPR 1D pulse sequence with water suppression was used to capture ^1^H-NMR spectra. The sequence was then processed using Bruker Topspin 3.2. The free induction decays (FIDs) obtained from the PFC were multiplied by a 0.3 Hz exponential line for broadening before fourier transformation. All NMR spectra were manually phased, baseline corrected, and chemical shift referenced to TSP (*δ* = 0.0) within MestReNova 12 (Mestrelab Research SL, Spain). The characteristic peaks of all metabolites were confirmed based on authority databases including the Human Metabolome Database (http://www.hmdb.ca/) and the Biological Magnetic Resonance Bank (http://www.bmrb.wisc.edu/metabolomics) [43, 44].

### 2.9. Enzyme-Linked Immunosorbent Assay (ELISA)

The serum was obtained from peripheral blood (PB) and the serum zonulin level [45] was measured using ELISA kits (Fankew, Shanghai FANKEL Industrial Co., Ltd., China) after a standard curve was developed to relate the concentration of zonulin.

### 2.10. Statistical Analysis

The Graphpad Prism 6 software (Version 6.01) was used to process and analyze the data in our experiment. All results were represented as *mean* ± *SEM*. One-way ANOVA was used to assess multiple groups' data such as weight, length of the colon, ELISA result, and the protein gray value of western blot. *P* < 0.05 was the cutoff as statistically significant. principal components analysis (PCA) and partial least-squares discriminant analysis (PLS-DA) were performed using SIMCA software 13.0 (Umetrics AB, Umea, Sweden). The differential metabolites were filtered by variable influence on projection (VIP) selection according to the PLS-DA with the filtering conditions of VIP > 1 and *P* < 0.05. The heat map was analyzed by Morpheus (https://software.broadinstitute.org/morpheus/).

## 3. Results

### 3.1. EA Prevented Colonic Shortening and Weight Loss in TNBS-Induced, IBD Rats

**Figure 1:**
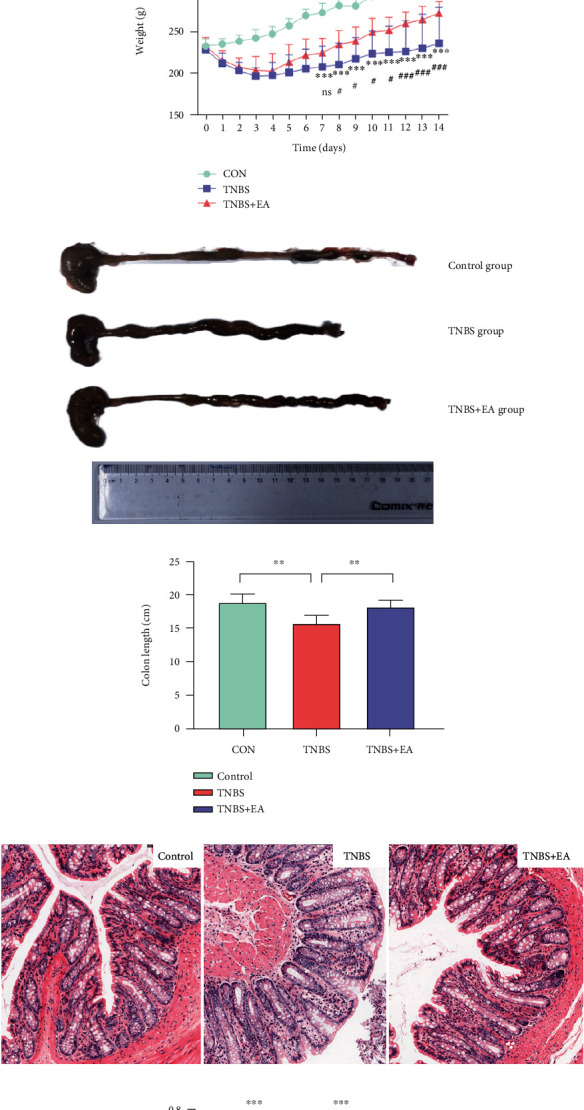
The experimental timeline and generic information regarding the subjects after treatment. (a) Experimental design (b) body weight comparisons (TNBS vs. Control: ∗*P* < 0.05; ∗∗*P* < 0.01; ∗∗∗*P* < 0.001. TNBS + EA vs. TNBS: ∗*P* < 0.05; ∗∗*P* < 0.01; ∗∗∗*P* < 0.001). (c, d) Colon lengths of (∗*P* < 0.05; ∗∗*P* < 0.01; ∗∗∗*P* < 0.001). (e) Hematoxylin and eosin staining of colon tissue at 14 days after TNBS treatment. (f) The mechanical threshold of visceral pain (∗*P* < 0.05; ∗∗*P* < 0.01; ∗∗∗*P* < 0.001). Values are expressed as mean ± SD (*n* = 8).

The degree of colonic shortening was found to be directly related to the severity of inflammation. As expected, the body weight of TNBS rats was significantly lower compared with that of the control group (*P* < 0.05; *P* < 0.01; *P* < 0.001.  Figure 1(b)). Compared to the TNBS group, the body weight of the TNBS + EA group rat was increased (*P* < 0.05; *P* < 0.01; *P* < 0.001Figure 1(b)), suggesting that treatment with EA was able to prevent colonic shortening and weight loss in IBD rats. The colon length in the TNBS group was significantly shorter than that in the control group (*P* < 0.01 Figures 1(c) and 1(d)).

### 3.2. EA Improves Colonic Morphology in IBD Rats

Severe colonic injury and inflammation were observed in TNBS-induced IBD rats, while the control group had ordered muscle architecture and clear intercellular space (Figure 1(e)). The IBD rat model showed destruction of mucosal architecture, incrassation of the muscle layer, cellular edema, and infiltration of immune cells (Figure 1(e)). However, EA-treated IBD subjects showed improved micromorphology compared to the untreated group (Figure 1(e)).

### 3.3. EA Stimulation Modulates the Pain Threshold in IBD Rats

The CRD test was used to determine the pain threshold, a marker for the severity of visceral pain. TNBS-treated rats had a higher pain threshold compared to the control group (*n* = 8, *P* < 0.001Figure 1(f)). However, the pain threshold was decreased in the TNBS + EA group (*P* < 0.001Figure 1(f)), indicating a lower level of visceral pain in EA-treated rats.

### 3.4. EA Treatment Affected PFC Metabolites in the TNBS Rat

**Figure 2:**
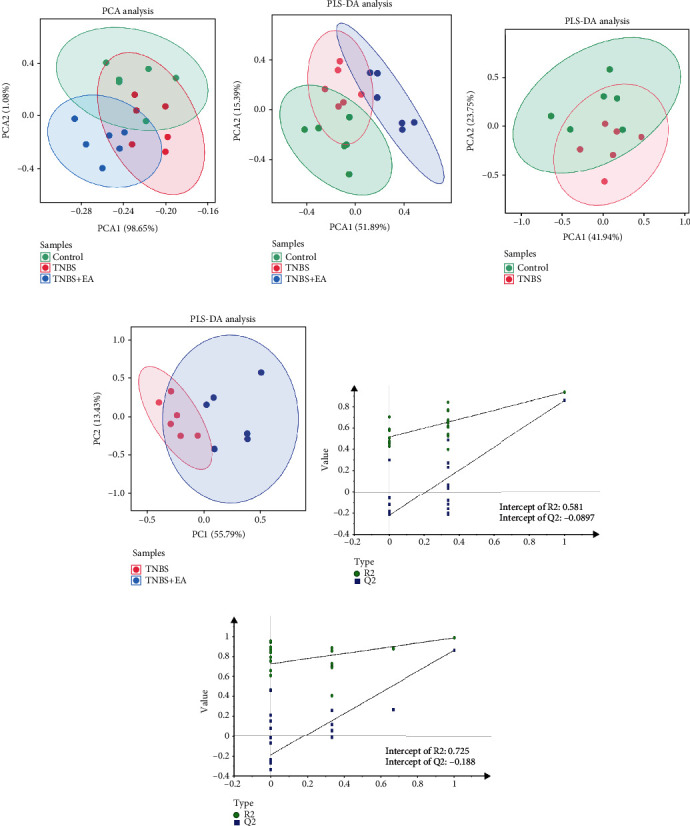
Analysis of metabolites in the PFC of IBD rats among groups (*n* = 6/group). (a) The PCA scatter plot between all groups. (b–d) The PLS-DA scatter plot among three groups (b: Control group, TNBS group, and TNBS + EA group. c: Control group and TNBS group. d: TNBS group and TNBS + EA group). (e, f) The parameters R2Y and Q2Y were determined by two PLS-DA scatter plots (e: Control group and TNBS group. f: TNBS group and TNBS + EA group).

To determine the effect of IBD on PFC metabolism, ^1^H-NMR spectroscopy was used to examine the neurochemistry in PFC tissue. A total of 15 metabolites were detected in our study. The PCA scatter plot showed differences among the control, TNBS, and TNBS + EA groups (Figure 2(a)). An unsupervised PLS-DA score plot was used to demonstrate these differences. This analysis revealed that the TNBS group had a distinct pattern from the control group (Figures 2(b)–2(d)). However, the difference was reduced following EA treatment. R2Y and Q2Y were used to confirm the two PLS-DA models (when both R2Y and Q2Y are equal to 1, the model is valid) (Figures 2(e) and 2(f)).

**Figure 3:**
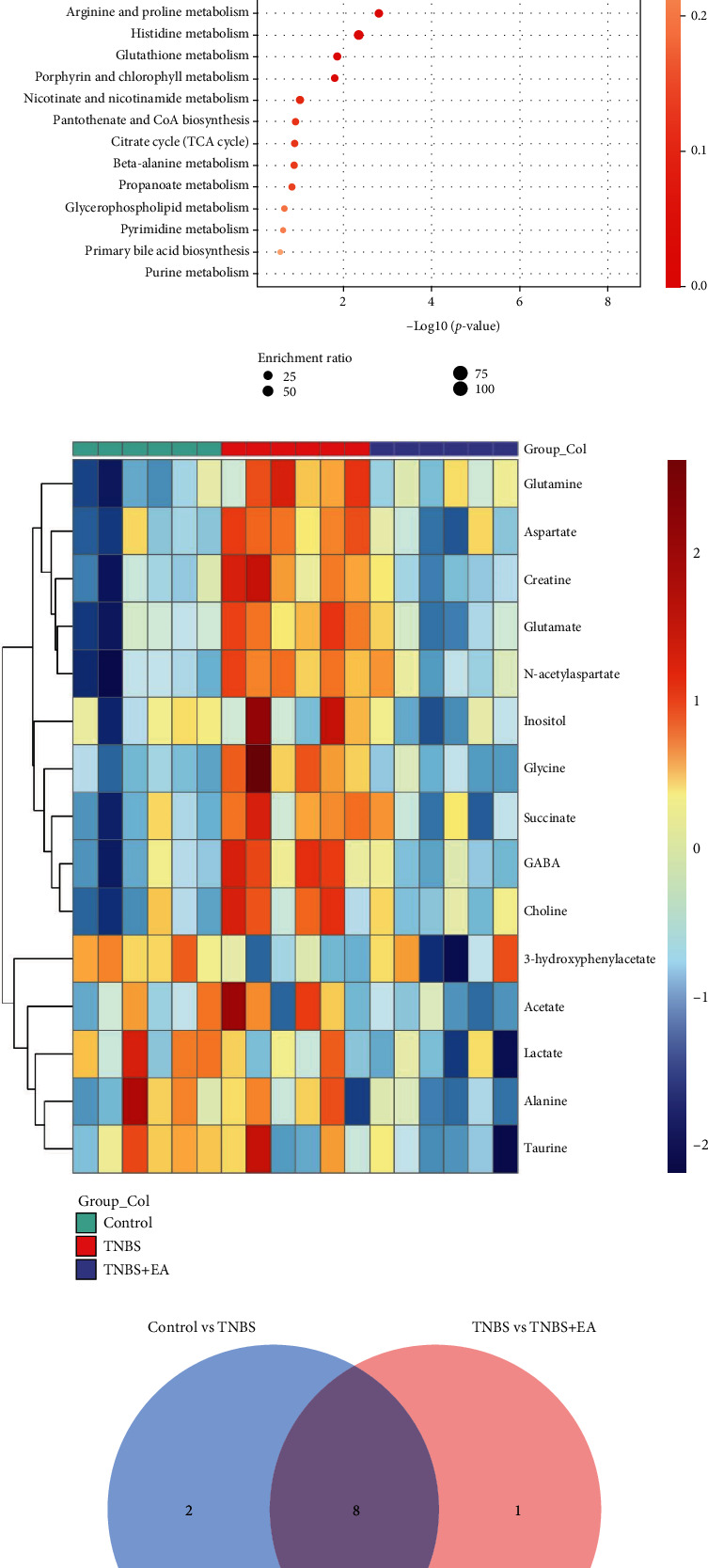
Analysis of metabolites in the PFC. (a, b) KEGG enrichment scatter plot of metabolites in the PFC (a: Control group vs. TNBS group; b: TNBS group vs. TNBS + EA group). (c) Heat map of the relative abundance of differential metabolites between the control group, TNBS group, and TNBS + EA group. Red represents a relatively high abundance in each sample, while blue represents a relatively low abundance. (d) The Venn diagram depicts the number of unique metabolites in the PFC between the TNBS vs. control group (blue) and TNBS + EA vs. TNBS group (pink) and their shared differential metabolites (violet). (e) The content differences of 8 metabolites were obtained by Venn diagram among the Control group, TNBS group, and TNBS + EA group.

A total of 14 compounds including inositol, creatine, taurine, lactate, aspartate, 3-hydroxyphenylacetate, GABA, alanine, choline, glycine, glutamate, glutamine, succinate, acetate, and N-acetyl aspartate were highlighted between the three groups. Different metabolites were analyzed under the following principles: *PLS* − *DA* *VIP* > 1, *P* < 0.05. Eleven metabolites were found to be unique between the control and TNBS groups. The KEGG pathway analysis of the 11 metabolites showed that 2 signaling pathways were significantly enriched (Figure 3(a)). Both pathways were associated with glutamate. Further, 8 different metabolites between the TNBS group and the TNBS + EA group were found to be altered. The analysis of the KEGG pathway showed that the 8 metabolites were also included in these two pathways (Figure 3(b)). In addition, the heat map showed concentration differences of these 15 different metabolites between the three groups (Figure 3(c)). Venn analysis showed that 8 metabolites were the key differentiators in the EA treatment of IBD rats (Figures 3(d) and 3(e)).

### 3.5. Effects of EA on Expressions of EAAT2, NMDA in PFC, and Expression of Zonulin in Serum

**Figure 4:**
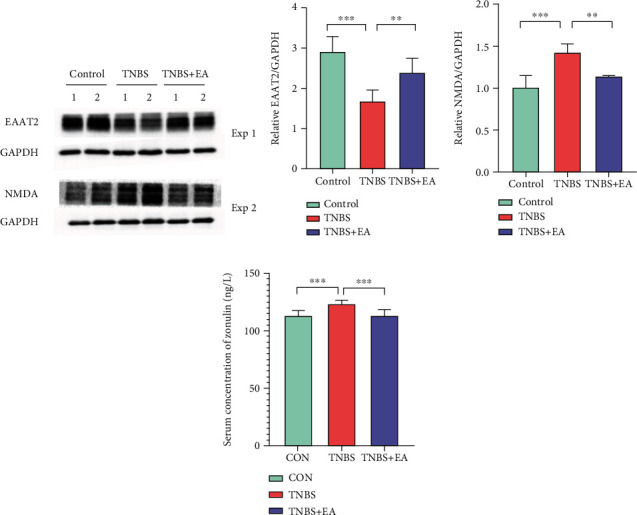
Effects of EA treatment on protein expression of EAAT2 (a, b) and NMDA (a, c) of the Control group, TNBS group, and TNBS + EA group (∗*P* < 0.05; ∗∗*P* < 0.01; ∗∗∗*P* < 0.001). Data were represented as mean ± SEM(*n* = 8). (d) The content difference of Zonulin in serum samples of three groups.

The result of the western blot showed that the expressions of excitatory amino acid transporters 2 (EAAT2) were significantly decreased (Figures 4(a) and 4(b)) and the expressions of NMDA were increased in the TNBS treatment group (Figures 4(a) and 4(c)). In contrast, EA treatment could reverse these changes. After EA treatment, the expression of zonulin in serum was decreased. The ELISA method was used to test the zonulin levels in rat serum. In our study, markedly enhanced zonulin levels were observed in IBD rats (Control vs. IBD: *P* < 0.01). After EA treatment, the content of zonulin in the serum was significantly reduced (TNBS vs. TNBS + EA: *P* < 0.001; Figure 4(d)).

### 3.6. Gut Microbiota Was Improved after EA Treatment

#### 3.6.1. Alpha Diversity and Beta Diversity

**Figure 5:**
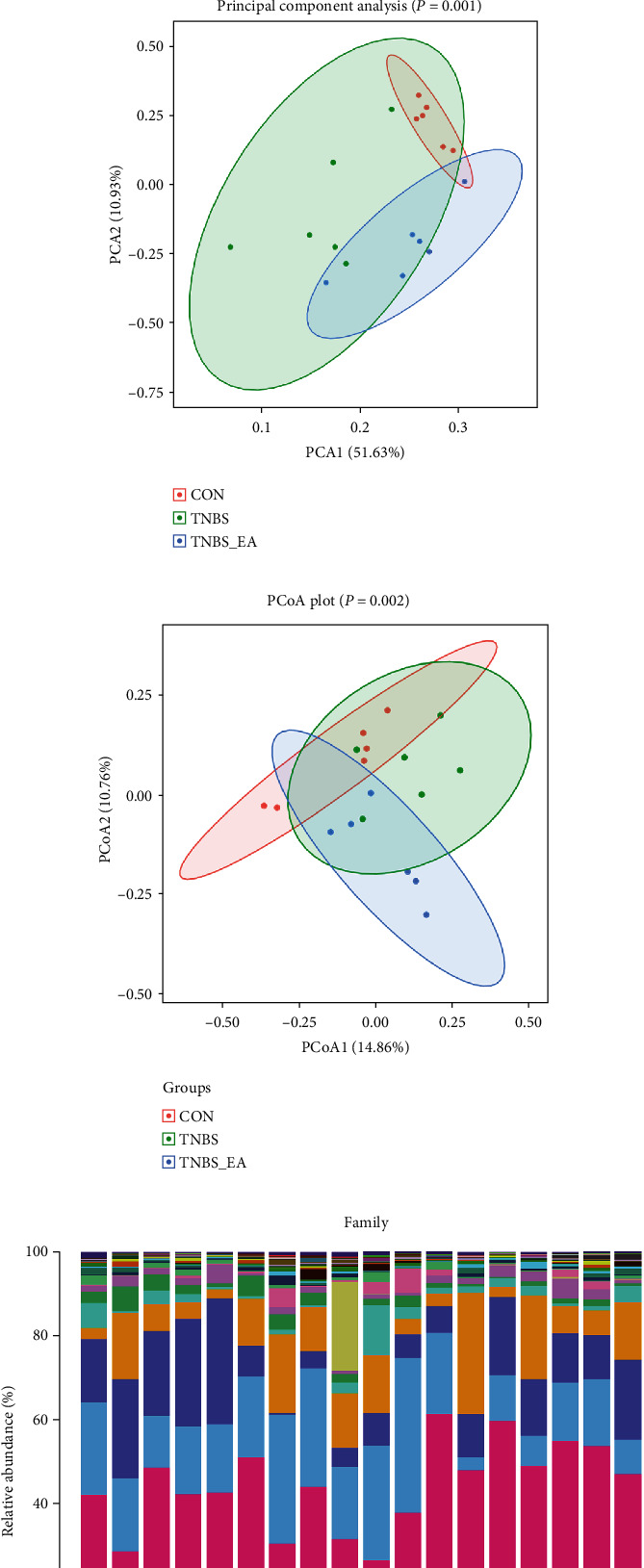
Analysis of differences in gut microbiota. Composition of gut microbiota in IBD rat. Violin plots showing alpha diversity results (a–d: Chao1, observed OTUs, Shannon's diversity, and Simpson's evenness) among three groups (control, TNBS, and TNBS + EA groups; *n* = 6/group). The data were assessed by a nonparametric Kruskal-Wallis test. (e, f) PCA and PCoA of networks beta-diversity was shown in the e and f. (∗*P* < 0.05; ∗∗*P* < 0.01). (g) The composition of gut microbiota in the Control group, TNBS group, and TNBS + EA group (*n* = 6/group). The taxonomic profiling of gut microbiota at the family level among three groups. (h) The Venn diagram depicts the number of different bacteria at the family level between TNBS vs. the control group (blue) and TNBS + EA vs. TNBS group (pink) and their shared differential bacteria (violet).

Compared to the control and TNBS + EA groups, a higher Shannon's diversity index, Simpson's evenness, chao1 species, and observed specie were observed in the TNBS group. However, there were no significant differences between each group (Figures 5(a)–5(d)). The principal coordinate analysis (PCoA) and principal components analysis (PCA) scatter plot showed that the gut microbiota of rats from the control group, TNBS group, and TNBS + EA group were separated at the genera level (Figures 5(e) and 5(f)). The results of alpha diversity showed that EA treatment did not change the richness and diversity of gut microbiota in TNBS mice. The results of beta diversity showed that the structure of gut microbiota in TNBS rats was changed after electroacupuncture treatment.

#### 3.6.2. Microbial Community Composition

**Figure 6:**
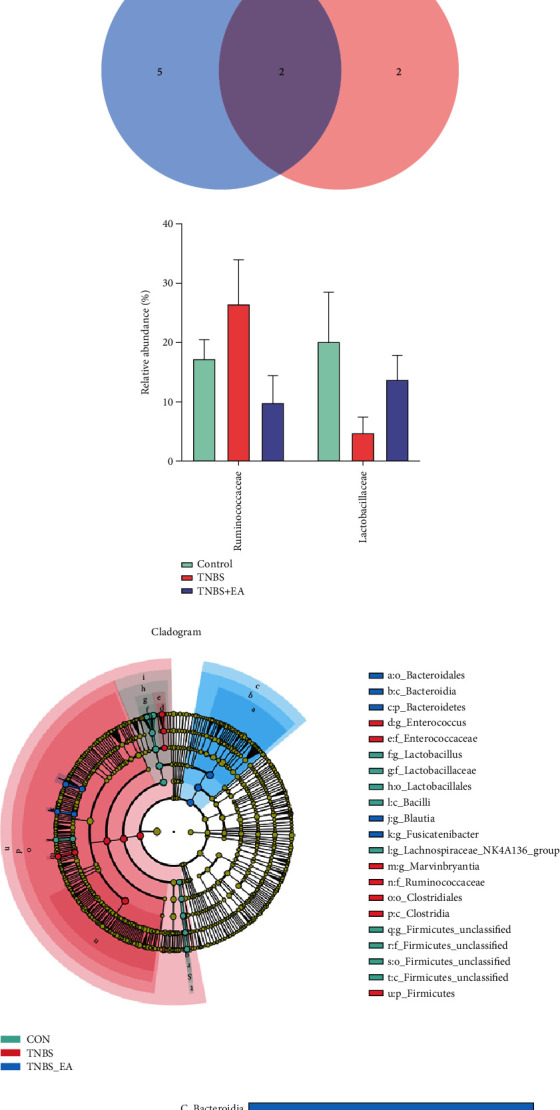
(a) Heat maps of the relative abundance among three groups. Red or blue represents the relative abundance in each sample. (b) The Venn diagram depicts the number of different bacteria at the family level between the TNBS vs. control group (blue) and TNBS + EA vs. TNBS group (pink) and their shared differential bacteria (violet). (c) Relative abundances of differential gut microbiota at the family level between each group (∗*P* < 0.05, ∗∗*P* < 0.01). (d, e) The LEfSe analysis of gut microbiota in three groups (Control, TNBS, and TNBS + EA group). The Cladogram generated by LEfSe analysis (d). Statistically significant taxa were screened according to LDA > 4 and *P* < 0.05 (e).

The microbial community composition of each group at the family level was shown in Figure 5(g). Compared to the control group, TNBS treatment increased the abundance of Ruminococcaceae, Enterococcaceae, Christensenellaceae, and Acidaminococcaceae, decreased the abundance of Lactobacillaceae, Peptostreptococcaceae, and Peptostreptococcaceae. The Venn diagram depicts the number of different bacteria at the family level between the TNBS vs. Control group (blue) and TNBS + EA vs. TNBS group (pink) and their shared differential bacteria (violet) (Figure 5(h)). A heat map was used to depict the differences between each group of the top 30 genera at the family level (Figure 6(a)). Venn analysis showed 2 differential bacteria between the control vs. TNBS group and TNBS vs. TNBS + EA group (Figure 6(b)). TNBS treatment increased the amount of Ruminococcaceae and decreased the amount of Lactobacillaceae. In contrast, EA treatment was shown to reverse these changes (Figure 6(c)).

The linear discriminant analysis effect size (LEfSe) (Figures 6(d) and 6(e)) demonstrated the gut microbial community composition of each group (*relative* *abundance* > 0.3%). Differential gut microbiota was selected with an LDA threshold greater than 4 and *P* < 0.05. The taxa with higher abundance in the control group were class Bacteroidia, order Bacteroidales, and phylum Bacteroidetes. The TNBS group showed a high abundance of the family Ruminococcaceae, class Clostridia, order Clostridiales, and phylum Firmicutes. TNBS + EA group showed a high abundance of the genus *Lactobacillus*, family Lactobacillaceae, order Lactobacillales, and class Bacilli.

### 3.7. Correlation Analysis of PFC Metabolites, PFC Protein Expression, and Gut Microbiomes

**Figure 7:**
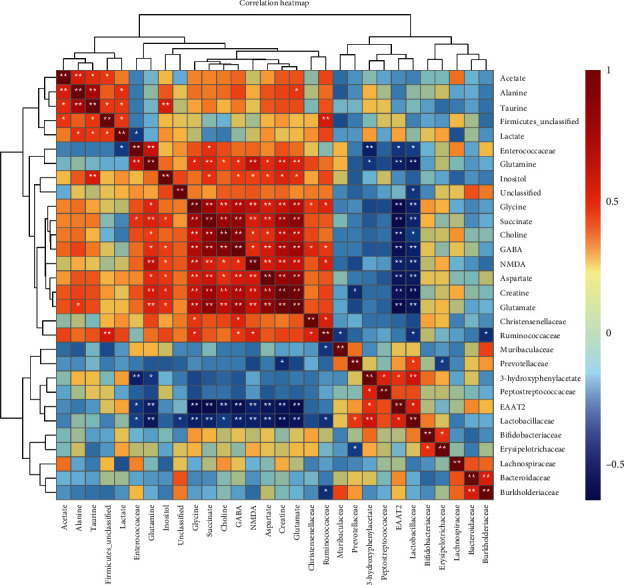
Correlation map of PFC metabolites, PFC protein expression, and gut microbial in TNBS rat. The red squares represent a positive correlation, while the blue squares represent a negative correlation (∗*P* < 0.05, ∗∗*P* < 0.01).

To further understand the effects of EA treatment on the brain-gut relationship of IBD rats, 14 metabolites, 2 glutamate-related proteins, and 14 gut microbes with the highest richness at the family level were analyzed. The results revealed a general correlation between various metabolites including glutamate in the PFC, NMDA, and EAAT2 protein in the PFC, and the gut microbiome. NMDAR, glutamate, and Ruminococcus were positively correlated with the pathological status of IBD rats, while EAAT2 and *Lactobacillus* were negatively correlated (Figure 7).

## 4. Discussion

We found that electroacupuncture therapy can relieve the visceral pain caused by IBD by regulating the glutamate energy system in the PFC. To determine whether EA can reduce visceral pain and improve the pathological state of IBD rats, a CRD test and hematoxylin-eosin staining were applied to assess the mechanical threshold and pathological changes in the intestine tissues. Patients with IBD have lower pain thresholds to intestinal stimulation, namely visceral hypersensitivity (VH) [46]. A lowered pain threshold (a higher VH) results in severer perceived pain in patients even with relatively little stimulation. In this study, the CRD test, which can measure the VH, was used to evaluate the intensity of visceral pain. Colonic distension was also used to assess visceral pain [47, 48].

It was found that EA greatly reduced visceral pain, regulated PFC's neurochemical metabolites, and restored the gut microbiome in TNBS-induced IBD rats. Compared with the TNBS group, EA-treated subjects showed higher body weights and longer colon lengths.

Current literature has demonstrated that EA can effectively treat IBD and relieve visceral pain symptoms. However, no study has clarified whether EA relieves visceral pain by modulating the CNS. Evidence has shown that the activation of PFC plays a key role in response to colorectal stimulation [36, 49, 50]. Thus, we measured metabolites in PFC to validate the effect of EA on visceral pain induced by IBD. The results showed that the level of glutamate in the PFC can be affected by IBD and the intervention of EA.

Glutamate is one of the most abundant and versatile excitatory amino acids in the CNS. It is mainly concentrated in the forebrain. As an important factor in nociceptive processing, glutamate plays a significant role in synaptic neurotransmission and neuronal growth, brain development, maturation, and synaptic plasticity [51]. Glutamic neurons would undergo excitatory toxicity when glutamate is overreleased under certain abnormal conditions, which eventually leads to cell death and is involved in the pathogenesis of many diseases including ischemia, hypoxia, trauma, and pathological pain [34].

The increase of extracellular glutamate can lead to the activation of glutamate ionic receptor N-methyl-D-aspartate receptor (NMDA), resulting in the long-lasting excitability of neurons [36]. Studies have shown that NMDA receptors are abundant in the PFC and are important in the mechanism of chronic pain [52]. In our study, TNBS treatment increased the content of glutamate and decreased the expression of NMDA receptors in PFC. However, EA treatment reversed those changes. These results suggest that EA can improve the visceral pain symptoms of IBD rats through glutamate modulation in the PFC.

Excitatory amino acid transporters (EAATs) make the main transport mechanism in the removal of extracellular glutamate in the CNS. EAATs consist of five transporters among which EAAT2 serves as the major glutamate transporter to neuroglial cells in PFC by removing extracellular glutamate [52–54], thereby relieving visceral pain caused by the activation of the NMDA receptor. As such, EAAT2 has been implicated in a variety of neurodegenerative diseases [55]. Some genetically related diseases, such as essential tremor (ET), are also affected by the reduced level of EAAT2 protein expression [56].

Previous studies have shown that electroacupuncture therapy has a protective effect on the nervous system by regulating the expression of EAAT2 protein [57]. EA treatment can alleviate cognitive impairment by regulating GLT1 in rodents (homologous to EAAT2 in humans) [58]. Therefore, it was reasonable to hypothesize whether electroacupuncture can also affect glutamate content by regulating the expression of EAAT2 protein and relieving visceral pain consequently. In our study, EAAT2 expression in the PFC was elevated in the TNBS + EA group in comparison to the TNBS group.

The gut microbiome can greatly affect the physiology of the host, including metabolic, trophic, and immune effects, therefore, its balance is of great importance in the overall health of the host [59, 60]. The concentration of *Lactobacillus* was greatly implicated in alleviating the intensity of visceral pain. As we expected, EA treatment was associated with an increased *Lactobacillus* concentration in IBD rats and relieved a range of inflammatory bowel disease symptoms, including visceral pain [61, 62].

Separately, the concentration of Ruminococcus correlated with the severity of visceral pain directly [63]. With high levels of intestinal barrier damage, Ruminococcus can easily reach the epithelial barrier and cause visceral pain [64]. The results obtained herein provided strong evidence to suggest that EA greatly increased the abundance of beneficial *Lactobacillus* while decreasing the content of harmful Ruminococcus and Marvinbryantia. Besides, we found that after EA treatment, the intestinal barrier function was significantly improved and glutamate content in the PFC of IBD rats was significantly reduced. Collectively, these results hint that EA may relieve pain by modulating the microbial gut-brain (MBG) axis.

The results of correlation analysis showed that there were obvious correlations among various metabolites including glutamate, EAAT2, and NMDA in the PFC, as well as various microorganisms in the intestinal tract. The decreased expression of EAAT2 in the PFC led to a decrease in its extracellular glutamate clearance ability. Extracellular glutamate can increase the stimulation of NMDA receptors, resulting in neurotoxicity which promotes visceral pain. The decrease of *Lactobacillus* and the increase of Ruminococcus also promoted the changes of metabolites in the PFC. The toxic effects of glutamate on the CNS system are extremely important in the occurrence of visceral pain. This study revealed that EA treatment was able to reduce the concentration of glutamate in the PFC and alleviate visceral pain in IBD rats. Verifying the mechanism of EA treatment on visceral pain is necessary to popularize the application of EA in IBD. In this study, we proved that glutamate, whose content can be quantitatively detected by magnetic resonance spectroscopy or other technical means, should be attached with great importance to such IBD-related pain. Accurate measurement of glutamate concentrate also helps to better evaluate the efficacy of EA treatment and is of great significance to improve the clinical therapeutic qualities.

The limitations of this study are mainly attributed to the use of ex vivo tissue. Therefore, future studies on the bowel function of IBD subjects should focus on examining the tissue in living subjects.

In summary, this study provided evidence to suggest that EA therapy can greatly reduce visceral pain in IBD rat models. It was first hypothesized and then confirmed that EA plays a role on the gut-brain axis through the modulation of associated metabolites in the PFC. We proposed that EA treatment can be applied to improve the intestinal as well as the immune function and restore the gut microbiome. Overall, the role of EA in treating IBD should be further explored for affected patients.

## Data Availability

All data for the conclusions in the evaluation paper are in the paper. Authors can be requested to provide relevant data upon request.
